# Unbiased retrieval of frequency-dependent mechanical properties from noisy time-dependent signals

**DOI:** 10.1016/j.bpr.2022.100054

**Published:** 2022-03-30

**Authors:** Shada Abuhattum, Hui-Shun Kuan, Paul Müller, Jochen Guck, Vasily Zaburdaev

**Affiliations:** 1 Max Planck Institute for the Science of Light, Erlangen, Germany; 2 Max-Planck-Zentrum für Physik und Medizin, Erlangen, Germany; 3 Biotechnology Center, Center for Molecular and Cellular Bioengineering, Technische Universität Dresden, Dresden, Germany; 4 Department of Biology, Friedrich-Alexander-Universität Erlangen-Nürnberg, Erlangen, Germany; 5 Max Planck Institute for the Physics of Complex Systems, Dresden, Germany; 6 Department of Physics, Friedrich-Alexander-Universität Erlangen-Nürnberg, Erlangen, Germany

## Abstract

The mechanical response of materials to dynamic loading is often quantified by the frequency-dependent complex modulus. Probing materials directly in the frequency domain faces technical challenges such as a limited range of frequencies, long measurement times, or small sample sizes. Furthermore, many biological samples, such as cells or tissues, can change their properties upon repetitive probing at different frequencies. Therefore, it is common practice to extract the material properties by fitting predefined mechanical models to measurements performed in the time domain. This practice, however, precludes the probing of unique and yet unexplored material properties. In this report, we demonstrate that the frequency-dependent complex modulus can be robustly retrieved in a model-independent manner directly from time-dependent stress-strain measurements. While applying a rolling average eliminates random noise and leads to a reliable complex modulus in the lower frequency range, a Fourier transform with a complex frequency helps to recover the material properties at high frequencies. Finally, by properly designing the probing procedure, the recovery of reliable mechanical properties can be extended to an even wider frequency range. Our approach can be used with many state-of-the-art experimental methods to interrogate the mechanical properties of biological and other complex materials.

## Why it matters

Fully understanding the response of a system that depends on the time scale of perturbation entails repetitive probing at different frequencies. However, when it comes to investigating the mechanical properties of a living cell or tissue actively responding to mechanical stress via biochemical signaling, repetitive tests are often unreliable. Here, we show how the frequency-dependent characteristics of a system can be accurately recovered from a noisy signal recorded while it responds to a time-dependent change in a single and fast measurement. This approach can dramatically upgrade existing and emerging high-throughput techniques by shortening measurement times and expanding the frequency range.

## Introduction

Interrogating the mechanical behavior of materials is of great significance for understanding the relation between their structure and function. For instance, the elastic behavior of a rubber band originates from entropic stretching of its constituent polyisoprene molecules. The shear-thickening properties of corn starch imply the existence of dynamically jammed structures (1), and the fluid-like viscous behavior of cellular aggregates can be linked to intermittent cell-cell interactions (2, 3, 4). Most materials, when observed at different time scales, will exhibit different mechanical behaviors. This phenomenon, attributed as viscoelasticity, has been studied extensively with the aim of unraveling complex mechanical properties and exploring novel materials (5). Characterizing mechanical properties has also been at the front line of biophysical research. Elucidating elastic and viscous properties of biological matter has led to significant insights into understanding cellular processes, morphogenesis, or the role of mechanical properties in disease (6, 7, 8).

Mechanical properties of materials are typically quantified via their stress (force per unit area [Pa]) - strain (relative displacement of the material [-]) relationship. To characterize the material response at different time scales, these properties are often represented in the frequency domain. The ratio of the Fourier-transformed stress $\hat{\sigma }\left(\omega \right)$ and strain $\hat{\varepsilon }\left(\omega \right)$ signals defines

(1) $$G^{\text{∗}}\left(\omega \right)=\frac{\hat{\sigma }\left(\omega \right)}{\hat{\varepsilon }\left(\omega \right)},$$

where ω is the angular frequency. The complex modulus $G^{\text{∗}}\left(\omega \right)$ is commonly used to describe the viscoelastic behavior of the materials:

(2) $$G^{\text{∗}}\left(\omega \right)=G^{\prime }\left(\omega \right)+iG^{\prime \prime }\left(\omega \right),$$

where $G^{\prime }$ and $G^{\text{″}}$ are the storage and the loss moduli, respectively.

Measurements of the mechanical properties in the frequency domain are routinely done in oscillatory rheometers but are time consuming and limited in accessible frequency ranges due to hardware constraints. Additionally, soft, living materials, such as single cells or tissues, are often too small for probing with traditional rheometers. The size constraints stimulated the development of probing techniques specifically for small length scales (in the range of nm to cm) such as particle microrheology (9,10), micropipette aspiration (11,12), atomic force microscopy (AFM) (13,14), optical stretching (15), and microfludic techniques (16,17). While measuring the mechanical properties in the frequency domain has been performed at small length scales, some materials measured change their properties as an active response when probed repetitively—a phenomenon known as mechanosensitivity (18, 19, 20). As this change can occur within seconds, applying even a few cycles of oscillatory measurements can lead to probing different mechanical behavior biased by this active response. Additionally, few of these techniques, such as AFM, suffer in the high frequency range from the effects of inertia and hydrodynamic drag (21), which narrows down the range of reliable frequencies that can be applied. Recent studies have shown that in the high frequency range, the viscous characteristics of cells dominate over the elastic characteristics. This behavior can be interpreted as a combined contribution of the viscous cytoplasm and the relaxation modes of individual cytoskeleton filaments (22,23). Thus, exploring the mechanical properties at high frequencies aids the investigation of the microscopic structural properties of cells that contribute to their mechanics. One approach to overcome these challenges is to perform single time-resolved measurements. Commonly, the time-dependent signals obtained are then fitted to a predefined model such as a Maxwell liquid, a Kelvin-Voigt solid, or a combination of both (24, 25, 26, 27, 28, 29). Fitting a model implicitly prescribes a certain viscoelastic behavior and limits the exploration of novel properties of specimens studied. To circumvent this, recent efforts have attempted to convert measurements conducted in the time domain to the frequency domain either directly (30) or after fitting to a preset function (31). The caveat, however, is that the measured signals are too complex to be described as a continuous analytical function and are usually accompanied with noise of different origins.

**Figure 1:**
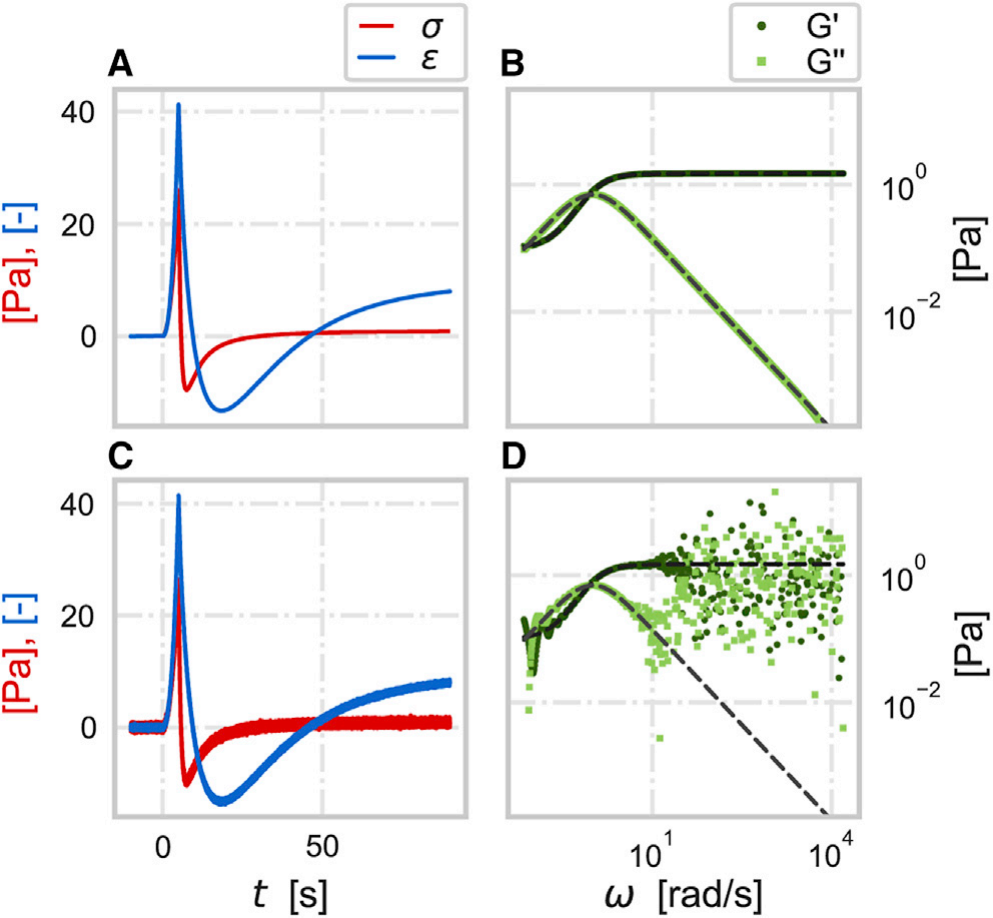
Viscoelastic behavior of a simulated standard linear solid material. (*A*) Ideal stress σ (red) and strain ε (blue) signals of an SLS material as a function of time. (*B*) Storage $G^{\prime }$ (dark green circles) and loss $G^{\text{″}}$ (light green squares) moduli of an SLS material calculated from the Fourier transforms of the signals in (*A*) via Eq. (1). (*C*) Stress σ and strain ε signals of an SLS material in time accompanied with random noise. (*D*) Storage $G^{\prime }$ and loss $G^{\text{″}}$ moduli of SLS material calculated from the Fourier transforms of the signals in (*C*). The dashed gray lines are the noise-free storage and loss moduli. The simulated SLS components are $E_{0}=$ 3/2 Pa and $E_{1}=$ 3/28 Pa for the springs and η=45/20Pa⋅s for the dashpot (see supporting material). The sampling frequency is 5000 Hz. The random noise has a zero mean and a standard deviation of 0.25 and 0.15 Pa for stress and strain signals, respectively.

Fig. 1 illustrates how even a moderate random noise added on top of the ideal standard linear solid (SLS) stress and strain signals (Fig. 1, *A* and *C*) dramatically changes the resultant frequency-dependent moduli (Fig. 1, *B* and *D*) calculated from Fourier-transformed signals using Eqs. (1) and (2) (see supporting material for details of simulations and motivation to the signal shape choice).

In this report, we present an unbiased approach to extract the complex modulus of materials from a single time-dependent signal without fitting a predefined model. Our approach utilizes the statistical properties of noise (zero mean and short-time correlation) and uses a modified Fourier transform with complex frequency to enhance the signal-to-noise ratio. Furthermore, we use our analysis pipeline to suggest an optimal time-dependent probing protocol, which can dramatically improve the quality of the frequency-dependent mechanical properties retrieved.

### Smoothing data with rolling average

One common method to reduce the effect of random noise is to average multiple independent measurements of the same sample. However, some materials, such as living biological samples, can exhibit a change in mechanical properties upon repetitive loading and, thus, multiple measurements are not feasible. This problem can be resolved by applying a rolling average to the time-dependent signal if the noise is short-time correlated (shorter than the sampling time) and has zero mean. Assume that an experimentally measured time-dependent signal can be represented as a sum of the true signal and random white noise ξ(t):

(3) $$\sigma_{\text{exp}}\left(t\right)=\sigma_{\text{true}}\left(t\right)+\xi \left(t\right).$$

The random white noise ξ(t) has a zero mean, ⟨ξ(t)⟩=0, and is uncorrelated, $\left\langle \xi \left(t\right)\xi \left(t^{\prime }\right)\right\rangle =C\delta \left(t-t^{\prime }\right)$, where $\delta \left(t-t^{\prime }\right)$ is the Dirac delta function and the constant *C* is the magnitude of the noise. Thus, the rolling average of the experimental values ${\bar{\sigma }}_{\text{exp}}\left(t\right)$ with a large enough averaging window should be statistically equal to the time-averaged true values ${\bar{\sigma }}_{\text{true}}\left(t\right)$ (here, we used the signal of stress as an example, but the same can be applied to the strain ε):

(4) $${\bar{\sigma }}_{\text{exp}}\left(t\right)\equiv \frac{1}{n+1}\overset{0}{\underset{j=-n}{\sum }}\sigma_{\text{exp}}\left(t+j\text{Δ}t\right)≃\frac{1}{n+1}\overset{0}{\underset{j=-n}{\sum }}\sigma_{\text{real}}\left(t+j\text{Δ}t\right),$$

where Δt is the sampling time of the experimental measurement, *n* is the number of time steps in the averaging window, and ${\bar{\sigma }}_{\text{exp}}\left(t\right)$ is the averaged signal. As is typical for the rolling average, the averaging window size should be chosen large enough to filter out noise but not too large to interfere with the true signal (see supporting material).

**Figure 2:**
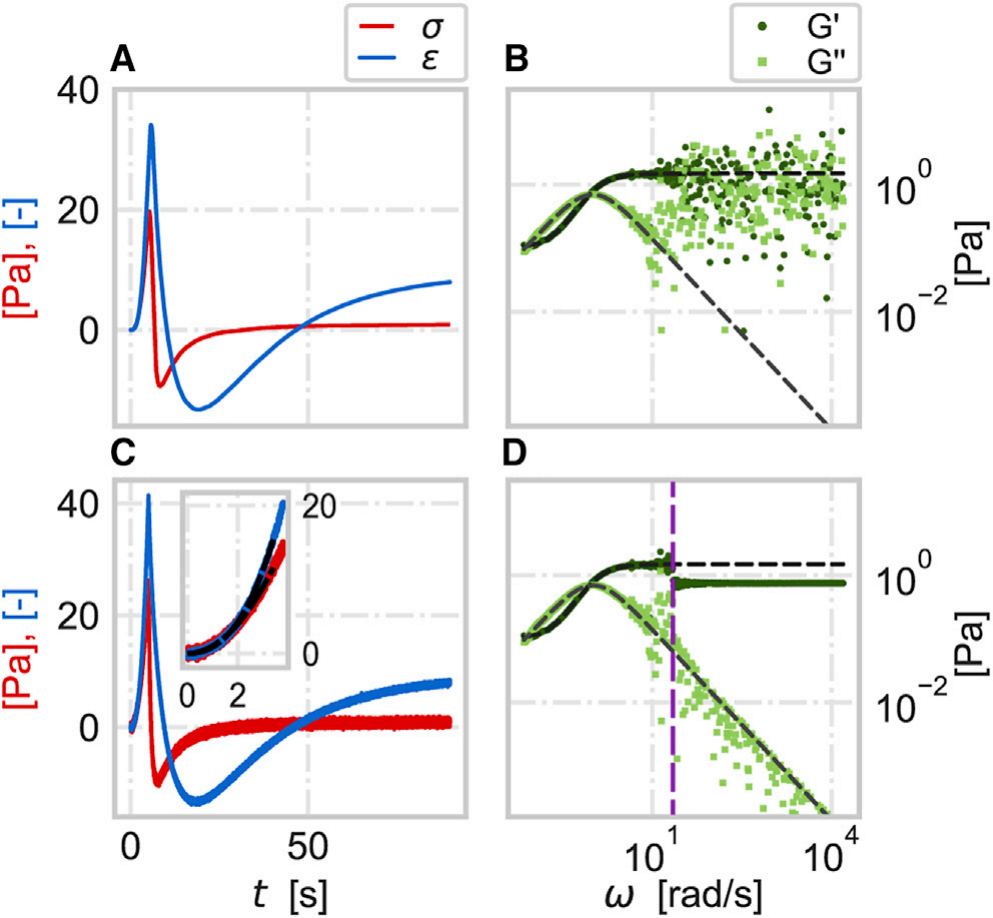
Recovery of viscoleastic properties from time-dependent signals accompanied with random noise. (*A*) Stress σ (red) and strain ε (blue) signals of an SLS material after applying a rolling average filter (*n* = 20,000). (*B*) Storage $G^{\prime }$ (dark green circles) and loss $G^{\text{″}}$ (light green squares) moduli of an SLS material calculated from the Fourier transforms of the averaged signals in (*A*) (Eq. (6)). (*C*) Stress σ and strain ε signals of an SLS material in time accompanied with random noise. The inset shows a fraction of the stress and strain signals and the corresponding sum of a polynomials fit (black). (*D*) Storage $G^{\prime }$ and loss $G^{\text{″}}$ moduli of an SLS material where the moduli at low frequencies (ω< 20 rad/s, indicated by the vertical purple line) are recovered from the moving average filtered stress and strain as shown in (*B*) and at high frequencies are recovered from the ratio of the truncated Fourier transforms (*z* = 2 s^-1^) of the fitted segments of stress and strain (up to *t*_*m*_ = 2.6 s) shown in the inset in (*C*). The dashed gray lines are the noise-free storage and loss moduli. The other simulation parameters of SLS are the same as in Fig. 1.

Importantly for our application, the Fourier transform (denoted with ˆ ) of the time-averaged signal ${\bar{\sigma }}_{\text{exp}}\left(t\right)$ can be linked to that of the true signal:

(5) $${\hat{\bar{\sigma }}}_{\text{exp}}\left(\omega \right)=\frac{1}{2\pi }\int_{-\infty }^{\infty }dt\;e^{-i\omega t}{\bar{\sigma }}_{\text{exp}}\left(t\right)≃\frac{{\hat{\sigma }}_{\text{true}}\left(\omega \right)}{n+1}\frac{e^{-in\omega \text{Δ}t}\left(1-e^{i\omega \left(n+1\right)\text{Δ}t}\right)}{1-e^{i\omega \text{Δ}t}},$$

(for the derivation, see supporting material). By applying a rolling average filter with the same window size for the noisy stress and strain signals (see Fig. 2 *A* for the averaged signals and *C* for the noisy signals before averaging shown for the example of SLS), the noise-free complex modulus can be calculated from the ratio of the Fourier transforms of the averaged signals:

(6) $$G_{\text{true}}^{\text{∗}}\left(\omega \right)=\frac{{\hat{\sigma }}_{\text{true}}\left(\omega \right)}{{\hat{\varepsilon }}_{\text{true}}\left(\omega \right)}≃\frac{{\hat{\bar{\sigma }}}_{\text{exp}}\left(\omega \right)}{{\hat{\bar{\varepsilon }}}_{\text{exp}}\left(\omega \right)}.$$

This averaging helps to properly recover the mechanical properties in the low frequency range (Figs. 1 *D* and 2 *B*; for a typical measured signal, the signal-to-noise ratio is often smaller in the high frequency range), but the higher frequencies are still problematic. To resolve this problem, we should first find out which part of the time-dependent signal affects the high frequency results and either make a better measurement or use some statistical method to enhance the signal-to-noise ratio in this part of the signal.

### Truncated Fourier transform

The short-time behavior of stress-strain signals has a strong effect on the moduli recovered in the high frequency range. For example, artificially shifting the origin of signals relative to each other by a small amount has a dramatic effect on the moduli recovered (see supporting material). However, transforming only the initial fraction of the signal from time to frequency domain is not possible with the conventional use of continuous or discrete Fourier transforms. Here, we propose to transform the initial fraction of the signal by truncating it via multiplication with an attenuating exponent $e^{-zt}$, where *z*, a non-negative real number, defines the inverse of the truncation time (we used the signal of stress as an example, but the same can be applied to the strain):

(7) $$\hat{\sigma }\left(\omega ,z\right)=\frac{1}{2\pi }\int_{0}^{\infty }dt\;\sigma \left(t\right)e^{-i\omega t-zt}.$$

The signal is assumed to start at t=0 (σ(t<0)=0). In fact, this expression might be viewed as a Fourier transform with a complex frequency, where the standard Fourier transform of the whole signal is recovered when $z\to 0^{+}$. The ratio R(ω,z) between the Fourier transform of the whole signal $\hat{\sigma }\left(\omega \right)$ and the Fourier transform of the truncated signal $\hat{\sigma }\left(\omega ,z\right)$ gives a measure of how close they are to each other and is written as

$$R\left(\omega ,z\right)≃\frac{\int_{0}^{t_{m}}dt\;\sigma \left(t\right)e^{-i\omega t-zt}}{\int_{0}^{\infty }dt\;\sigma \left(t\right)e^{-i\omega t}}=\frac{\int_{0}^{t_{m}}dt\;\left(a_{0}+a_{1}t+a_{2}t^{2}+\cdots \right)e^{-i\omega t-zt}}{\int_{0}^{\infty }dt\;\left(a_{0}+a_{1}t+a_{2}t^{2}+\cdots \right)e^{-i\omega t}}=\frac{a_{0}\frac{1-e^{-\left(i\omega +z\right)t_{m}}}{\left(i\omega +z\right)}+a_{1}\frac{1-e^{-\left(i\omega +z\right)t_{m}}(1+(i\omega +z)t_{m}))}{{\left(i\omega +z\right)}^{2}}+\cdots }{\frac{a_{0}}{i\omega }-\frac{a_{1}}{\omega^{2}}+\cdots }.$$

If the measurement time is much larger than the truncation time, $t_{m}\gg z^{-1}$, we can substitute the infinite integration limit in Eq. (7) by $t_{m}$. Additionally, we use the polynomial expansion $\sigma \left(t\right)=\sum_{j=0}^{\infty }a_{j}t^{j}$ without losing any generality (the polynomial expansion suggests the signal has a well-defined Taylor expansion around t=0, which, in general, is true in almost all signals). Finally, in the limit, $zt_{m}\to \infty$, the above ratio R(ω,z) becomes

(9) $$R\left(\omega ,z\right)≃\frac{\frac{a_{0}}{i\omega +z}+\frac{a_{1}}{{\left(i\omega +z\right)}^{2}}+\cdots }{\frac{a_{0}}{i\omega }-\frac{a_{1}}{\omega^{2}}+\cdots },$$

suggesting that it is close to 1 if ω≫z. This indicates that the Fourier transform of the data in a constrained range ($t<t_{m}$) can provide an accurate estimate of the Fourier-transformed signal for high frequencies. In other words, unlike most other analysis methods, using the Fourier transform with complex frequency does not require the signals to reach a steady state (as, e.g., in (30)), as the signal beyond t≫1/z will not affect the value of the Fourier-transform results significantly. The value *z* then naturally sets the lower limit of frequencies when Eq. (7) is close to the normal Fourier transform. Combined with the criterion $zt_{m}\gg 1$, we can naturally link this lower bound to the time of the last signal measurement $t_{m}$. In practical terms, the value of *z* can be determined by setting $e^{-zt_{m}}\ll \delta$, where δ is a threshold value that determines the attenuation strength of the signal after $t_{m}$.

Interestingly, the numerator (the argument works for the denominator as well) of the ratio in Eq. (9) indicates that the signal at high frequencies is dominated by the lower-order terms in the polynomial expansion. This suggests that the partial polynomial fitting, which can enhance the signal-to-noise ratio in a certain, limited range of the signal, can lead to accurate high-frequency results even if the signal cannot be represented in its entirety by a finite polynomial expansion (thus, the accuracy of the smaller-order terms determines the upper bound of the frequency; see supporting material). Fig. 2 *D* shows the complex shear modulus of an SLS material where the mechanical properties at lower frequencies (ω< 20 rad/s for *z* = 2 s^-1^) are recovered using a rolling average. The moduli at higher frequencies (ω≫z) are calculated by applying the Fourier transform with truncation (*z* = 2 s^-1^) to the fitted (by polynomial up to degree j=5) fraction of the noisy signal (see inset in Fig. 2 *C*; see supporting material). The slight discrepancy between the true and the recovered complex modulus is associated with the quality of the fit. Increasing the number of the fitted measurement points improves the fit quality. In the study of Kwon (31), the whole signal is fitted with a polynomial sum. This, however, leads to inaccuracies, especially for complex signal shapes. Thus, reducing the shape complexity of the signal and using the truncated Fourier transform, which can be applied on signals that cannot be transformed with the standard Fourier transform, will result in higher-quality fits (see supporting material for more details and also the effect of the different noise levels). This naturally leads to the most forward-looking aspect of this work.

### Optimized probing protocol

Combining the idea of the order expansion and the lower bound definition $z=-\text{log}\left(\delta \right)/t_{m}$, a more accurate restoration of the complex modulus can already be influenced by choosing an optimized measurement protocol. Although it is common to perturb the material by fast probing and slow relaxation (a step function) for enhancing the signal-to-noise ratio (the Fourier transform of the linear probe is $\left|\int_{0}^{\infty }dt\;bt\;\exp^{-i\omega t}\right|=\left|\frac{b}{\omega^{2}}\right|$, and the magnitude increases as the slope *b* increases), a slower probing can gather more reliable data to be used with the truncated Fourier transform. The corresponding fitting of the slower measurement can lead to a better signal-to-noise ratio due to more sampled data. Indeed, in some methods, either the stress or the strain can be set by the user (for example, an AFM or a rheometer), and by choosing a predefined signal that perturbs the material in a simple manner, the fitting becomes easier and more accurate.

**Figure 3:**
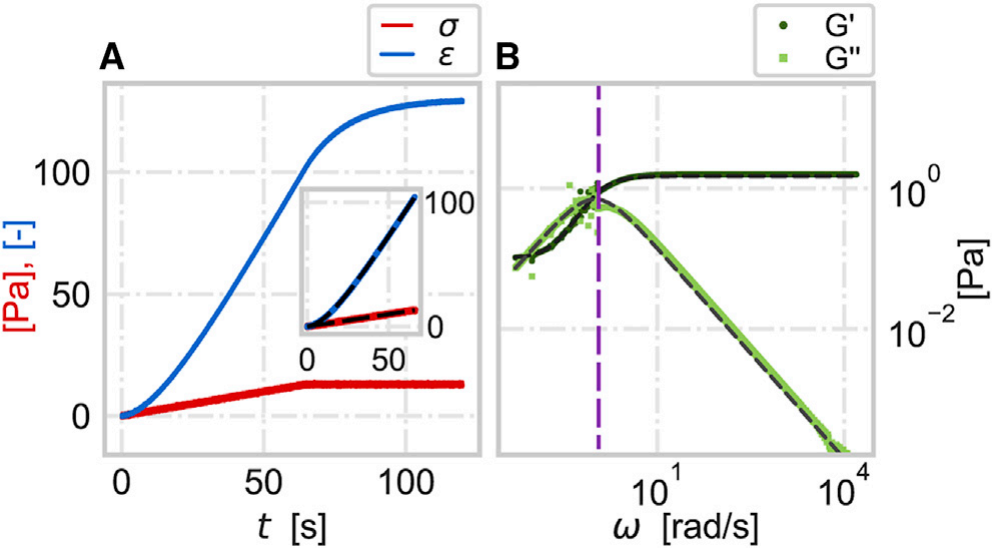
Alternative probing protocol. (*A*) Simulated SLS material probed with linear stress σ=At (red) and its strain response ε (blue). The inset shows a fraction of the stress and strain signals and the corresponding sum of polynomials fit (black). (*B*) Storage $G^{\prime }$ (dark green circles) and loss $G^{\text{″}}$ (light green squares) moduli of an SLS material where the moduli at low frequencies (ω< 1 rad/s, indicated by the vertical purple line) are recovered from the moving average (*n* = 20,000) filtered stress and strain and at high frequencies are recovered from the ratio of the truncated Fourier transforms (*z* = 0.4 s^-1^) of the fitted segments up to *t*_*m*_ = 66 s) of stress and strain shown in the inset in (*A*). The dashed gray lines are the noise-free storage and loss moduli. The other simulation parameters of SLS are the same as in Fig. 1.

In Fig. 3 *A*, a linear stress σ(t)=At is applied to the SLS material, where *A* is a positive constant. The stress signal is then fitted with a linear function while the strain is fitted with the summation of polynomials (in this example, up to the fourth order). The advantage of using a linear stress ramp becomes clear when applying the truncated Fourier transformation on the fitted fraction of the signal. In this case, the fitting process is simplified due to the linearity of the signal and the larger number of data points that can be included in the fit. To confirm that our method works for a wide range of material properties, we verified the method using other mechanical models, such as Kelvin-Voigt, standard linear fluid, and power law material (see supporting material). The complex shear modulus of the material is remarkably well recovered in the lower frequency range (below the purple line in Fig. 3 *B*) with the rolling average method and at the higher frequencies with the truncated Fourier transform (Fig. 3 *B*).

### Experimental validation

**Figure 4:**
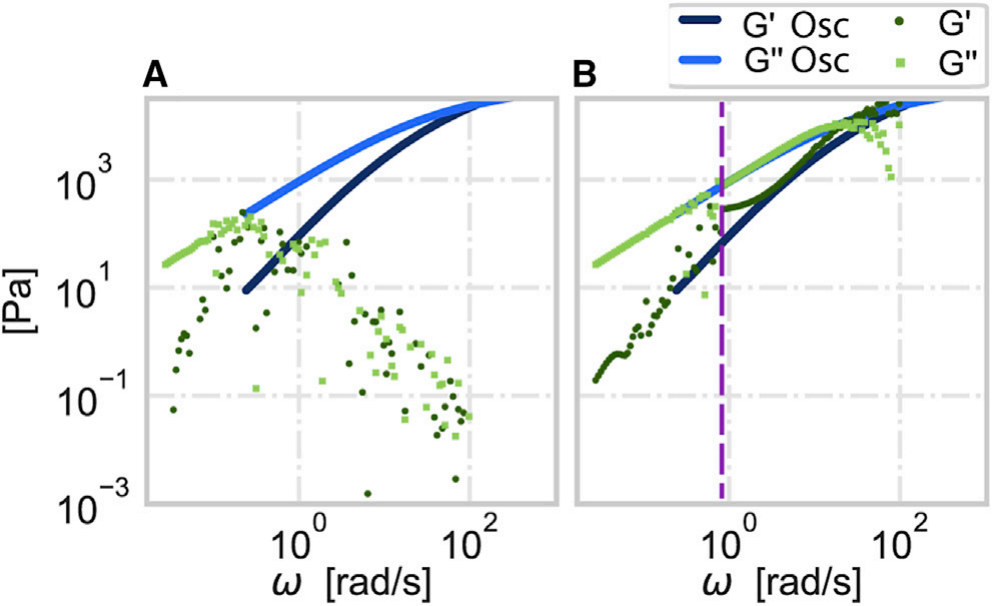
Mechanical properties of silicone fluid measured with rheometer. Storage $G^{\prime }$ (dark blue) and loss $G^{\text{″}}$ (light blue) moduli were recovered directly from the oscillatory stress and strain measurements. (*A*) Storage $G^{\prime }$ (dark green circles) and loss $G^{\text{″}}$ (light green squares) moduli calculated directly from the ratio of the Fourier-transformed time-dependent linear stress and the corresponding strain signals. (*B*) Storage $G^{\prime }$ (dark green circles) and loss $G^{\text{″}}$ (light green circles) moduli recovered in the low frequency range (below the vertical purple line) using a rolling average filter (n = 120)) and at high frequencies from the ratio of the truncated Fourier transforms of the fitted fraction of the applied linear stress and the corresponding strain signals (see supporting material). For this data, the fraction (up to *t*_*m*_ = 57 s) was fitted with a summation of polynomials up to the ninth order. The purple line is considered the lower useful limit of the truncated Fourier transformation approach (*z* = 0.26 s^-1^).

Although the field of rheology has been explored for many decades, finding a viscoelastic material that can be used for validation of the method is not a straightforward task, as the properties could change depending on the technique (32). Thus, to demonstrate the practical applicability of our approach to measured data, we used the rheometer, a gold-standard method in the field of rheology, to probe the mechanical properties of a precharacterized viscoelastic material, silicone fluid (AK 1000000, Tsukuba, Wacker, Japan) (see supporting material). A rheometer is typically used for mechanical characterization due to its ability to apply oscillatory stress and strain signals and thus directly extract the frequency-dependent loss and storage moduli. Furthermore, using the same device, time-dependent strain or stress measurements can also be performed. Here, we applied a linear stress signal and measured the strain signal of the silicone fluid (see supporting material). Fig. 4 *A* depicts the storage and loss moduli of the silicone fluid measured from the oscillatory stress and strain signals, as well as the moduli calculated from the Fourier transforms applied directly to the linear stress and the corresponding strain signals. It is evident that the moduli cannot be properly retrieved and can even be erroneously assumed to decrease for higher frequencies, if relying directly on the Fourier transforms of the noisy signals. Fig. 4 *B* shows, in addition to the moduli from the oscillatory measurement, the storage and loss moduli calculated from a combination of the rolling average (for frequencies below the purple line) and the truncated Fourier transform applied to the signals fitted (for frequencies above the purple line). Comparison of the mechanical properties recovered from both approaches shows a dramatic improvement in the recovery of $G^{\prime }$ and $G^{\text{″}}$ in both low and high frequency ranges.

**Figure 5:**
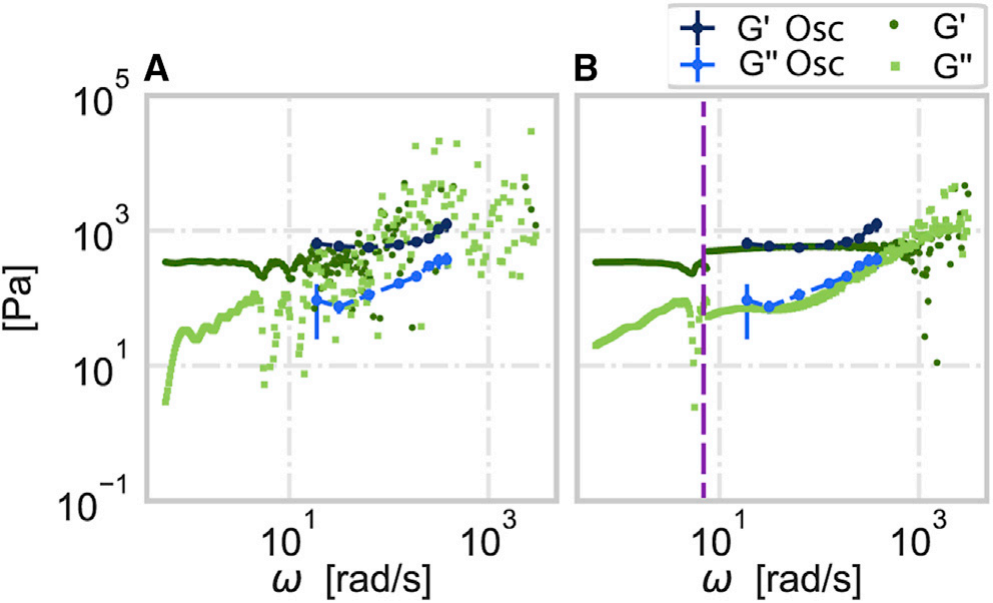
Mechanical properties of low-gelling point agarose hydrogel measured with AFM. Storage $G^{\prime }$ (dark blue circles) and loss $G^{\text{″}}$ (light blue circle) moduli were recovered directly from the oscillatory indentation and force measurements, and the error bars indicate the standard deviation of 27 measurements. (*A*) Storage $G^{\prime }$ (dark green circles) and loss $G^{\text{″}}$ (light green squares) moduli calculated directly from the ratio of the Fourier-transformed time-dependent linear indentation and the corresponding force signals. (*B*) Storage $G^{\prime }$ (dark green circles) and loss $G^{\text{″}}$ (light green circles) moduli recovered in the low frequency range (below the vertical purple line) using a rolling average filter (*n* = 2000) and at high frequencies from the ratio of the truncated Fourier transforms of the fitted fraction of the applied linear indentation and the corresponding force signals. For this data, the fraction (up to *t*_*m*_ = 1.8 s) was fitted with a summation of polynomials up to the sixth order. The purple line is considered as the lower useful limit of the truncated Fourier transformation approach (*z* = 5 s^-1^).

To demonstrate that our method is also applicable for micron-sized length scales, we used an AFM to probe a low-gelling point agarose hydrogel (Sigma-Aldrich, Hamburg, Germany) with a slow linear indentation (velocity 1 μm/s) using a PNP-TR-TL (Nanoworld, Neuchâtel, Switzerland) cantilever (nominal spring constant of 0.08 mN/m) modified with 5 μm diameter polystyrene beads (microParticles, Berlin, Germany) (see supporting material). The force F(t) and indentation δ(t) signals are then used to calculate the complex shear modulus using the relation originating from the Hertz model for a parabolic indenter (33) and the elastic-viscoelastic correspondence principle

(10) $$G^{\text{∗}}=\frac{3}{8}\frac{1-\nu }{R^{\frac{1}{2}}}\frac{\hat{F}\left(\omega \right)}{\hat{\text{Δ}}\left(\omega \right)}.$$

where $\hat{F}\left(\omega \right)$ and $\hat{\text{Δ}}\left(\omega \right)$ are the Fourier transforms of F(t) and $\delta^{\frac{3}{2}}\left(t\right)$, respectively, and ν is the Poisson’s ratio of the material. In addition to the linear indentation measurement, we applied, also using the AFM, an oscillatory measurement to directly extract the complex shear modulus. Fig. 5 shows in blue the storage (dark blue) and loss (light blue) moduli evaluated from the oscillatory measurements where the error bars indicate the standard deviation and in green the storage and loss moduli calculated from the time-dependent signals. Fig. 5 *A* compares the results of the oscillatory measurements to the complex modulus retrieved from applying the Fourier transform directly to the force and indentation signals. The mechanical properties, especially at high frequencies, are heavily affected by the noise. Applying our method to the time-dependent signals improved greatly the precision of the retrieved mechanical properties as shown in Fig. 5 *B*. Due to the hydrodynamic-drag effects on the cantilever when oscillating in high frequency, the frequency range of reliable oscillatory measurements is narrower than the range retrieved using our method. Additionally, since the oscillatory measurement is time consuming in the lower frequency range, we limited the applied frequency to 3 Hz to keep the measurement duration in the range of few minutes. Here, we show that method can be also used for materials measured in the small length scale and can extend the range of reliable mechanical properties to a wider range of frequency.

## Conclusions

We provide an unbiased and model-free method to recover the frequency-dependent material properties from noisy time-dependent stress and strain signals from a single measurement. The effect of random uncorrelated noise can be removed from the lower frequency range by a rolling average filter, and the partial fitting combined with the truncated Fourier transform extends the region of reliable retrieval of mechanical properties to the higher frequency range. To further improve the quality of data recovered, we propose the use of an alternative measuring protocol in which the sample is probed rather slowly, allowing for accurate fitting of a larger fraction of the data with a polynomial series. Unlike the standard Fourier transforms, the truncated Fourier transform does not require knowledge about the behavior of stress and strain at very long times (30). This makes the truncated Fourier transform together with the partial fitting ideal for a variety of measured signals that do not reach a steady state or cannot be transformed with a discrete Fourier transform (e.g., square and triangular signals). Still some assumptions need to be considered for our method. First, the noise accompanying to the experimental data is treated as random white noise. This assumption excludes other noise types including thermal drift. In the case of rheometer and AFM measurements presented here, this assumption seems to be sufficient when compared with measurements directly performed in the frequency domain. Moreover, it would be interesting to theoretically consider more complex noises in future work. Second, the fitting of the initial fraction of the signal becomes more accurate with an increasing number of fitted polynomials. This, however, causes the fit to be more sensitive to noise. With our derivation, we show that the lower-order polynomials dominate the fitting in the higher frequency. Thus, it would be adequate to only rely on the fitting of lower-order polynomials. One possible extension of our method would relate to the estimation of the non-linearity of biological materials. In such case, ramps with different rates can be used for probing the change in the mechanical properties of such materials. Applying our theoretical approach to existing experimental techniques enhances not only the analysis of time-dependent measurements but also promotes the investigation of wider ranges of time and length scales. Finally, the approach of converting time-dependent signals to the frequency domain proposed here is of course not limited to mechanical probing but may well find beneficial application in many other areas of physics where this is required.

## Author contributions

S.A., H.-S.K., J.G., and V.Z. conceived the project. S.A. and H.-S.K.. developed and designed the methodology and created the models. S.A. carried out the experiments and the data analysis. S.A. and P.M. implemented the algorithms. S.A., H.-S.K., J.G., and V.Z. wrote the original draft. S.A., H.-S.K., P.M., J.G., and V.Z. reviewed and edited the manuscript. J.G. and V.Z. secured the funding.

## Declaration of interests

The authors declare no competing interests.

## Supporting citations

References (34,35) appear in the supporting material.

## Supporting material

Document S1. Figures S1–S7

Document S2. Article plus supporting material
