# Supplementary material for: Unbiased retrieval of frequency-dependent mechanical properties from noisy time-dependent signals
Source: Biophys Rep (N Y). 2022 Mar 30;2(3):100054. doi: 10.1016/j.bpr.2022.100054 (PMC9680806; doi:10.1016/j.bpr.2022.100054)
Supplement: Document S1. Figures S1–S7 [file mmc1.pdf]

Biophysical Reports, Volume *2*

Supplemental information

Unbiased retrieval of frequency-dependent mechanical properties from noisy time-dependent signals

Shada Abuhattum, Hui-Shun Kuan, Paul Müller, Jochen Guck, and Vasily Zaburdaev

Supplemental Material

Unbiased retrieval of frequency-dependent mechanical properties from noisy time-dependent signals

Shada Abuhattum,[1,2,3,^] Hui-Shun Kuan,[2,4,5,^] Paul Müller,[1,2,3] Jochen Guck,[1,2,3,6] and Vasily Zaburdaev[2,4,5,*]

[1]*Max Planck Institute for the Science of Light, 91058 Erlangen, Germany*
[2]*Max-Planck-Zentrum für Physik und Medizin, 91058 Erlangen, Germany*
[3]*Biotechnology Center, Center for Molecular and Cellular Bioengineering, Technische Universität Dresden, 01307 Dresden, Germany*
[4]*Department of Biology, Friedrich-Alexander-Universität Erlangen-Nürnberg, 91058 Erlangen, Germany*
[5]*Max Planck Institute for the Physics of Complex Systems, 01187 Dresden, Germany*
[6]*Department of Physics, Friedrich-Alexander-Universität Erlangen-Nürnberg, 91058 Erlangen, Germany*

I. MATERIALS AND METHODS

A. Standard Linear Solid (SLS) data generation

Standard linear solid model Fig. S1 consisting of two springs ($E_0 = 3/2\,\text{Pa}$, $E_1 = 3/28\,\text{Pa}$) and one dashpot ($\eta = 45/28\,\text{Pa}\cdot\text{s}$) was used for generating the time-dependent stress and strain signals.

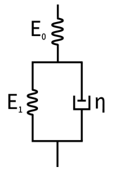

Figure S1: Illustration of the standard linear solid model (SLS model).

The stress-strain equation of the SLS model is:

$$\sigma + \frac{\eta}{E_0 + E_1}\dot{\sigma} = \frac{E_0 E_1}{E_0 + E_1}\varepsilon + \frac{\eta E_0}{E_0 + E_1}\dot{\varepsilon} \qquad (1)$$

where σ and ϵ are the stress and the strain, respectively. The equation was solved numerically using Python for the following time-dependent stress signal for Fig. 1 and 2 in the main text:

$$\sigma(t) = \begin{cases} t^2, & t \leq 5\,\text{s} \\ (5^8 + \frac{2\cdot 5^9}{7})\frac{1}{t^6} - \frac{10\cdot 5^4}{7}\frac{1}{t^2} + 1, & 5\,\text{s} < t \leq 90\,\text{s} \end{cases}$$

and the following for Fig. 3 in the letter:

$$\sigma(t) = \begin{cases} \frac{t}{5}, & t \leq 65\,\text{s} \\ 13, & 65\,\text{s} < t \leq 120\,\text{s} \end{cases}$$

The sampling rate was set to 5000 Hz and the random noise with a zero mean and a standard deviation of 0.25 Pa and 0.15 was added to the stress and strain signals, respectively. The main goal behind using an artificially generated dataset is to obtain a ground truth that can be compared to the results of our method. The signal profile is different than the simple signals typically used for rheological measurement. However, such complex profiles can be experimentally obtained in various methods, for example shear stress profiles in microfluidic channels [1]

B. Silicone Fluid Rheology

We measured the mechanical properties of silicone fluid - AK 1000000 (Wacker, Japan) with MCR502 Rheometer (Anton Paar, Austria). We placed a small portion of the fluid (500 units/mL) between two parallel plates with a diameter of 20 mm and applied a frequency sweep measurement in the range of 0.025−40 Hz. The duration time of the oscillatory method ranged from 15− 18 mins. Then we performed a time dependent measurement by applying a linear stress ramp with a slope of $\frac{1}{6}$ Pa/s as shown in Fig. S2.

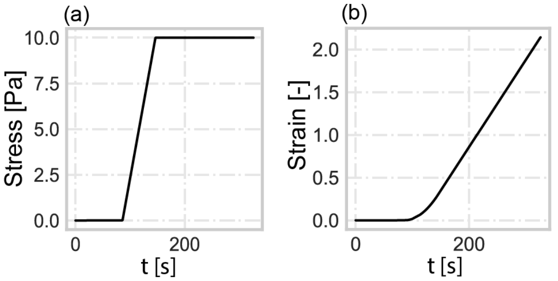

Figure S2: Rheological measurement of silicone fluid. (a) Time-dependent stress signal applied using the rheometer on a silicon fluid sample between parallel plates. (b) The corresponding strain signal.

^ These two authors contributed equally
* vasily.zaburdaev@fau.de

C. AFM measurements of agarose

The agarose hydrogels were probed in two different measurement modes using the AFM; oscillatory and linear indentation. The hydrogels were prepared by dissolving low-gelling-point agarose (Sigma-Aldrich, Germany) in warmed ddH_2O to a final concentration of 0.5 %. While the solution was still warm, 35 µl were added to an ethanol cleaned foil, then a glass cover slip was placed on top. After 30 min at room temperature the agarose hydrogels were crosslinked. The foil was then removed and the hydrogels were kept for 24 hours with PBS for reaching steady state. For all AFM measurements, a PNP-TR-TL cantilever (Nanoworld) with a nominal spring constant of 0.08 mN/m was modified by gluing to the tip a 5 µm diameter polystyrene bead (microparticles GmbH, Berlin).

For the linear indentation measurements, the piezoactuator was moved with a velocity 1 µm/s until a force of 4 nN was reached. The length of linear indentation movement was approximately 14 s. The stress $F(t)$ and indentation $\delta(t)$ signals were recorded. For calculating the complex modulus, the relation which originates from the Hertz model for a parabolic indenter [2] and the elastic-viscoelastic correspondence principle was used:

$$G^* = \frac{3}{8}\frac{1-\nu}{R^{\frac{1}{2}}}\frac{\hat{F}(\omega)}{\hat{\Delta}(\omega)}. \tag{2}$$

Here $\hat{F}(\omega)$ and $\hat{\Delta}(\omega)$ are the Fourier transforms of $F(t)$ and $\delta^{\frac{3}{2}}(t)$, respectively, and ν is the Poisson's ratio of the material.

For the oscillatory measurements, we followed the method described in [3]. Briefly, prior to the measurement, the hydrodynamic drag function $b(h)$ of the cantilever was evaluated from the non-contact oscillations of the cantilever in the medium at different distances h from the bottom surface. The hydrodynamic drag coefficient $b(0)$ was then extracted by extrapolating the function to distance 0 from the sample. For this cantilever the coefficient was $b(0) = 5.28$ µNs/m. For probing the mechanical properties of the hydrogel, the cantilever was first lowered with a velocity of 10 µm/s until an indentation of $\delta_0 \approx 1$ µm was reached, then a sinusoidal movement of the piezoactuator with a small amplitude 10 nm for a period of 10 cycles was applied to the hydrogel in a range of frequencies of $3 - 60$ Hz. The recorded force F and indentation δ signals were then fitted with sinus function and their amplitude (A_F and A_δ) and phase shifts (φ_F and φ_δ) were extracted for every frequency. The complex modulus of the material was calculated from the Hertz model linearized due to small perturbations and corrected for the effect of the hydrodynamic drag:

$$G^* = \frac{1-\nu}{4\sqrt{R\delta_0}}\left(\frac{\hat{F}(\omega)}{\hat{\delta}(\omega)} - i\omega b(0)\right). \tag{3}$$

where $\hat{F}(\omega)$ and $\hat{\delta}(\omega)$ are the Fourier transforms of $F(t)$ and $\delta(t)$, respectively, and are calculated as follows:

$$\frac{\hat{F}(\omega)}{\hat{\delta}(\omega)} = \frac{A_{F(\omega)}e^{i\varphi_{F(\omega)}}}{A_{\delta(\omega)}e^{i\varphi_{\delta}(\omega)}} \tag{4}$$

The hydrogel was probed in 27 different locations where the length of the measurement for every location was $t \approx 2$ min.

D. Fourier transform and fitting method

For performing the Fourier transform calculations for the discrete signals we used the method described in [4] or the python implementation of the discrete Fourier transform. For fitting a sum of polynomials (order < 9) to a fraction of the time-dependent signal we used `lmfit` package in Python.

II. THE GENERAL AVERAGING METHOD

A set of experimental time-dependent data $\sigma_{exp}(t)$ can be written as:

$$\sigma_{\rm exp}(t) = \sigma_{\rm true}(t) + \xi(t), \tag{5}$$

where $\sigma_{true}(t)$ is the noise-free data, $\xi(t)$ is the time-dependent noise, and $\sigma_{true}(t < 0) = 0$ denotes the onset of the physical perturbation at $t = 0$. For simplicity, the noise is chosen as the white noise which has zero mean $\langle\xi(t)\rangle = 0$ and zero correlation $\langle\xi(t)\xi(t')\rangle = C\delta(t - t')$, where C is the strength of the noise and $\delta(t)$ is the Dirac delta function.

Then, the noise can be reduced by summing the vicinity data (the averaging method). The averaged data $\bar{\sigma}_{exp}(t)$ is written as:

$$\bar{\sigma}_{\rm exp}(t) = \frac{1}{n+1}\sum_{j=-n}^{0}\sigma_{\rm exp}(t + j\Delta t)f(j\Delta t), \tag{6}$$

where Δt is the data sampling frequency,n is the averaging window size and $f(t)$ is the averaging filter. In the main text, we chose $f(t) = 1$ which indicates that each data point is equally important.

The corresponding Fourier transform of the averaged data then is written as:

$$\begin{aligned}\hat{\bar{\sigma}}_{\rm exp}(\omega) &= \frac{1}{n+1}\sum_{j=-n}^{0}\int_0^\infty dt\, e^{-i\omega t}\sigma_{\rm exp}(t + j\Delta t)f(j\Delta t) \\ &= \frac{1}{n+1}\sum_{j=-n}^{0}\int_{j\Delta t}^\infty d\tau\, e^{-i\omega(\tau - j\Delta t)}\sigma_{\rm exp}(\tau)f(j\Delta t) \\ &= \sum_{j=-n}^{0}\frac{\hat{\sigma}_{\rm true}(\omega)f(j\Delta t)}{n+1}e^{i\omega j\Delta t} + \sum_{j=-n}^{0}\frac{\hat{\xi}_j(\omega)f(j\Delta t)}{n+1},\end{aligned} \tag{7}$$

where $\int_{j\Delta t}^{\infty} d\tau\, e^{-i\omega\tau}\sigma_{\rm true}(\tau) = \int_0^{\infty} d\tau\, e^{-i\omega\tau}\sigma_{\rm true}(\tau)$ due to $\sigma_{\rm true}(\tau<0)=0$, and $\int_0^{\infty} dt\, e^{-i\omega t}\xi(t+j\Delta t) = \hat{\xi}_j(\omega)$. Because of the properties of the white noise, statistically $\hat{\xi}_j(\omega)$ behaves the same with different j index, and $\sum_j \hat{\xi}_j(\omega) = 0$.

If the averaging filter eliminates the noise $\sum_{j=-n}^{0} f(j\Delta t)\xi(t+j\Delta t) = 0$, the Fourier transform of the noise-free data can be derived from the Fourier transform of the averaged data:

$$\hat{\sigma}_{\rm true}(\omega) = \frac{\hat{\bar{\sigma}}(\omega)}{\sum_{j=-n}^{0} \frac{f(j\Delta t)}{n+1} e^{i\omega j\Delta t}}. \quad (8)$$

In the main text, we used the simplest filter $f=1$, and the equation (8) can be written as:

$$\hat{\sigma}_{true}(\omega) = \frac{\hat{\bar{\sigma}}(\omega)(1-e^{i\omega\Delta t})(n+1)}{e^{-in\omega\Delta t}\left(1-e^{i\omega(n+1)\Delta t}\right)} \quad (9)$$

In Fig. S3 we tested the effect of the averaging window size on the results of the complex modulus. The window size is the smallest at the top row and the largest at the bottom row. The results of the complex modulus became more accurate with increasing the window size especially in the low frequency range. Increasing the window size to even larger values will lead to loss in the signal features.

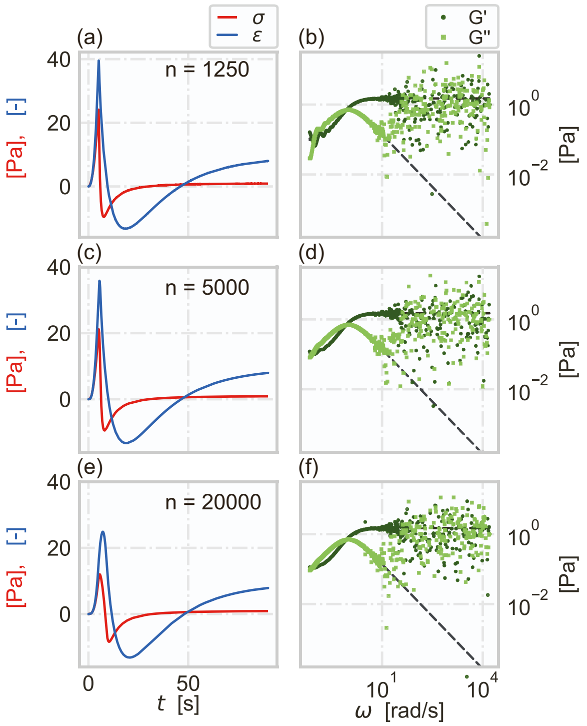

Figure S3: Averaging with different window sizes. The left column shows the averaged stress σ (red) and strain ϵ (blue) signals with different window sizes (a) $n=1250$, (b) 5000, and (c) 20000. The right column shows the storage G' (dark green circles) and loss G'' (light green squares) moduli calculated from the ratio of the Fourier transforms of the corresponding averaged stress and strain signals on the left. The dashed gray lines are the noise-free storage and loss moduli. The other simulation parameters of are the same as in Fig. 1 in the main text

III. STRONG EFFECT OF TIME SHIFT IN STRESS-STRAIN DATA

To illustrate that a signal at short times has strong effect on the recovered mechanical properties, we shift the stress and strain time-dependent signals (without noise) for an SLS material by 40 data points relative to each other (corresponding just to 8 ms) (Fig. S4 (a)). The corresponding complex modulus graphs show the drastic change in the high frequency range resulting from the shift of the signals in time (Fig. S4 (b)).

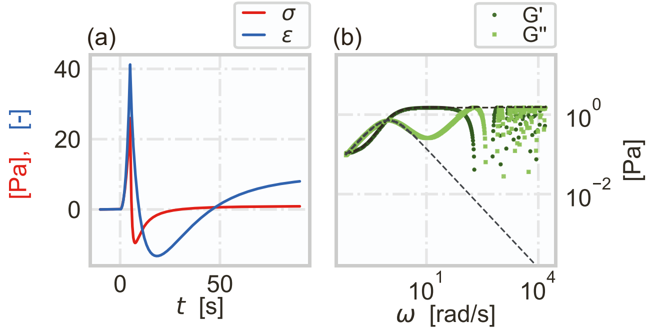

Figure S4: Shifting time-dependent data. (a) Stress σ (red) and strain ϵ (blue) signals of an SLS material shifted by 40 data points relative to each other. (b) Storage G' (dark green circles) and loss G'' (light green squares) moduli of SLS material calculated from the Fourier transforms of the signals in (a) via Eq. (1) in the main text. The dashed gray lines are the noise-free storage and loss moduli. The simulated SLS components are $E_0 = 3/2\,\mathrm{Pa}$ and $E_1 = 3/28\,\mathrm{Pa}$ for the springs and $\eta = 45/28\,\mathrm{Pa}\cdot\mathrm{s}$ for the dashpot (see supplemental information). The sampling frequency is 5000 Hz.

IV. THE SIGNAL-TO-NOISE RATIO AT HIGH FREQUENCIES

For a given noise-free time-series signal $\sigma(t)$, where $\sigma(t<0)=0$, without losing any generality, it can always be written as a power series:

$$\sigma(t) = a_0 + a_1 t + a_2 t^2 + \cdots . \tag{10}$$

Assume the signal starts at time $t=0$, the corresponding Fourier transform is written as:

$$\begin{aligned}\sigma(\omega) &= \frac{1}{2\pi}\int_0^\infty \left(a_0 + a_1 t + a_2 t^2 + \cdots\right) e^{i\omega t}\, dt \\ &= \frac{1}{2\pi}\left(\frac{a_0}{i\omega} + \frac{a_1}{(i\omega)^2} + \frac{2a_2}{(i\omega)^3} + \cdots\right).\end{aligned} \tag{11}$$

This indicates the Fourier transformed signal $\sigma(\omega)$ is dominated by the lower order of the polynomial expansion at the high frequencies. If the noise of the signal has zero mean and is short-time correlated, such as the Gaussian white noise, the Fourier transform is constant at all frequencies. Thus, the signal-to-noise ratio of the Fourier transformed noisy signal is becoming smaller for higher order polynomial expansion terms for higher frequencies.

V. COMPLEX MODULUS AT HIGH AND LOW FREQUENCIES

The retrieval of the complex modulus from the noisy data is performed by using the rolling average method for the low frequency range and the truncated Fourier transform on a fitted fraction of the data for the high frequency range. Fig. S5 (a) and (b) shows the complex moduli obtained using the rolling average and the truncated Fourier transform methods, respectively. The rolling average method recovers the complex modulus at the low frequency range while the truncated Fourier transform recovers it at the high frequency range. Thus, we combine the results of the two methods as shown in Fig. S5 (c), where the frequency that separates the two methods (in this specific case $\omega = 20\,\mathrm{rad/s}$) is selected to ensure that the condition, $\omega \gg z$ discussed in Eq. (9) in the main text, is fulfilled. Specifically, in the main text, two conditions are mentioned: 1) $e^{-zt_m} \ll \delta$ or, equivalently, $zt_m \gg 1$, and 2) $\omega \gg z$. While z has to be as small as possible, its product with maximal measurement time should be still very large. Then the approximation would be valid for frequencies much larger than z. In practice, the parameters can be chosen by the following argument. Identify a frequency where the reconstructed moduli start to exhibit strong fluctuations (For example, $\omega = 20\,\mathrm{rad/s}$ in Fig. S5 or Fig. 2 in the main text), choose a value of z that is smaller than ω and fulfills the condition $e^{-zt_m} \ll \delta$, where $\delta < 0.05$ (For Fig. 2, $t_m = 2.6\,\mathrm{s}$, $z = 2\,\mathrm{s}^{-1}$ and $e^{-zt_m} = 0.005$).

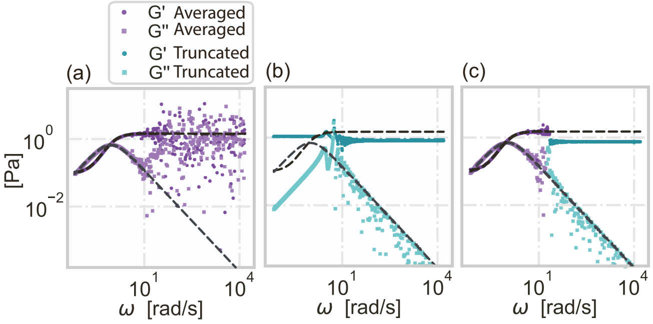

Figure S5: Combining the complex moduli. (a) Storage G' and loss G'' moduli (purple) of SLS material calculated from the Foruier transform of the averaged signals in Fig. 2(a) in the main text. (b) Storage G' and loss G'' moduli (cyan) of the same material calculated from the truncated Fourier transforms of the signals in Fig. 2(c) in the main text. (c) Combining the storage G' and loss G'' moduli of both methods, where the frequency that separates the two methods ($\omega = 20\,\mathrm{rad/s}$) is set to be significantly higher than the value of ($z = 2\,\mathrm{s}^{-1}$). The dashed gray lines are the noise-free storage and loss moduli. The other simulation parameters of SLS are the same as in Fig. 1 in the main text.

VI. NOISE LEVEL EFFECT ON THE COMPLEX MODULUS

We evaluated the effect of the signals' noise level on the retrieval of the complex modulus. We defined the signal to noise ratio (SNR) as the ratio between the mean of the signal and the standard deviation of the noise. Each row of Fig. S6 shows stress and strain signals with different SNR levels (a) no noise, (b) SNR = 100 and (c) SNR = 10. The middle and right columns show the corresponding complex modulus calculated using a standard Fourier transform and using our method, respectively. Although both methods show similar complex modulus results for clean data, the standard Fourier transform appears very sensitive to noise and fails in retrieving the characteristics of the SLS material for signals accompanied with noise. On the other hand, the complex modulus calculated with our method matches the noise free complex modulus even in significantly large noise levels (SNR = 10).

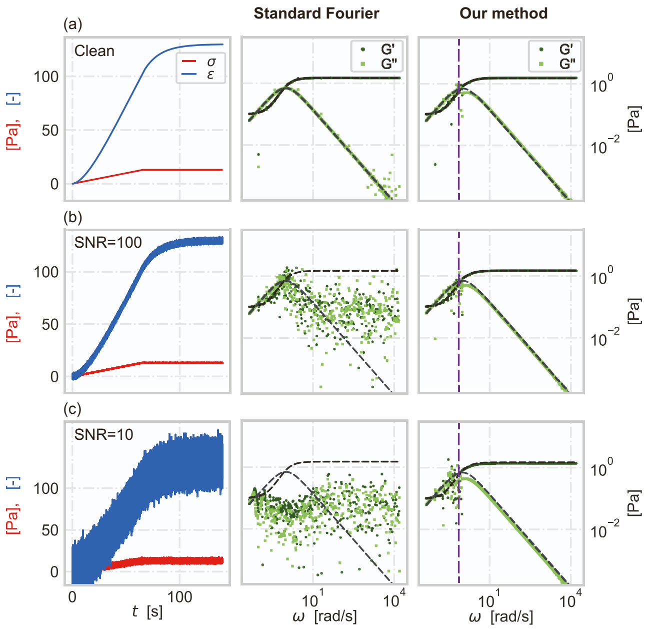

Figure S6: Effect of noise level on the complex modulus of simulated SLS material. Each row shows on the left stress (red) and strain (blue) signals with different noise levels; (a) no noise, (b) SNR = 100 and (c) SNR = 10. The storage G' (dark green circles) and loss G'' (light green squares) moduli calculated from the corresponding stress and strain signals on the left using the standard Fourier transform (middle column) and the rolling average (below the purple line) combined with the truncated Fourier transform (above the purple line) (right column). The dashed gray lines are the noise-free storage and loss moduli. The other simulation parameters of SLS are the same as in Fig. 1 in the main text.

VII. A RANGE OF MECHANICAL PROPERTIES

To confirm that our method is valid for materials with a wide range of mechanical properties, we applied it on Kelvin Voigt, standard linear fluid (Jeffrey's model), and power law (represented with a fractional spring-pot element [5, 6]) models probed with a linear strain function:

$$\varepsilon(t) = \begin{cases} \frac{t}{5}, & t \leq 20\,\mathrm{s} \\ 4, & t > 20\,\mathrm{s} \end{cases}$$

The stress and the strain signals for each model were accompanied with random noise. Fig. S7 shows that the mechanical properties of each of the models is retrieved when using our method. The power law model, specifically, shows some artifacts in the loss modulus G'' at the high frequency range. Due to the fact that the power law model is non-linear and more complex when compared with spring-dashpots models, it requires fitting higher order polynomials (here for all the models, the fraction of the stress signals shown in the inset was fitted with summation of polynomials up to the 11th order). Fitting polynomial summation up to higher orders can be computationally expensive and more sensitive to noise. Still, the overall trend of a power law material is profoundly retrieved.

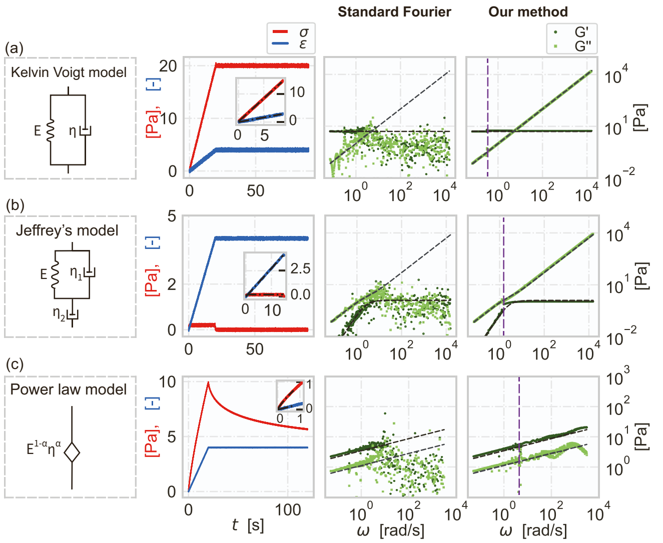

Figure S7: Retrieval of a wide range of mechanical properties represented with three different mechanical models; (a) Kelvin Voigt model, (b) standard linear fluid (Jeffery's model), and (c) power law model represented by a fractional model with the spring-pot element. Each row shows, in the four panels from left to right; (1) a sketch of the model, (2) the stress σ (red) and strain ε (blue) signals with a random noise accounting for an SNR $= 100$ and an inset showing the fitted fraction of the signals, (3) the storage G' (dark green circles) and loss G'' (light green squares) moduli calculated from the corresponding stress and strain signals on the left using the standard Fourier transform, and (4) the same moduli retrieved with the rolling average (below the purple line) combined with the truncated Fourier transform (above the purple line). The dashed gray lines in the last two panels are the noise-free storage and loss moduli. The values for all the models were $E = 5\,\mathrm{Pa}$ and $\eta, \eta_1, \eta_2 = 1\,\mathrm{Pa}\cdot\mathrm{s}$ and for the power law model $\alpha = 0.2$.

SUPPORTING REFERENCES

[1] Fregin, B., F. Czerwinski, D. Biedenweg, S. Girardo, S. Gross, K. Aurich, and O. Otto, 2019. High-throughput single-cell rheology in complex samples by dynamic real-time deformability cytometry. *Nature communications* 10:1–11.

[2] Hertz, H., 1881. Über die Berührung fester elastischer Körper. *J. Die Reine Angew. Math* 92:156–171.

[3] Alcaraz, J., L. Buscemi, M. Grabulosa, X. Trepat, B. Fabry, R. Farré, and D. Navajas, 2003. Microrheology of human lung epithelial cells measured by atomic force microscopy. *Biophysical Journal* 84:2071–2079.

[4] Evans, R., M. Tassieri, D. Auhl, and T. A. Waigh, 2009. Direct conversion of rheological compliance measurements into storage and loss moduli. *Physical Review E* 80:012501.

[5] Schiessel, H., R. Metzler, A. Blumen, and T. Nonnenmacher, 1995. Generalized viscoelastic models: their fractional equations with solutions. *Journal of physics A: Mathematical and General* 28:6567.

[6] Bonfanti, A., J. L. Kaplan, G. Charras, and A. Kabla, 2020. Fractional viscoelastic models for power-law materials. *Soft Matter* 16:6002–6020.
